# Is thermogenesis really needed for brown adipose tissue–mediated metabolic benefit?

**DOI:** 10.1172/JCI159296

**Published:** 2022-05-02

**Authors:** Jin-Seon Yook, Shingo Kajimura

**Affiliations:** Division of Endocrinology, Diabetes and Metabolism, Beth Israel Deaconess Medical Center and Harvard Medical School, Boston, Massachusetts, USA. Howard Hughes Medical Institute, Chevy Chase, Maryland, USA.

## Abstract

Brown adipose tissue (BAT) dissipates energy in the form of heat and functions as a metabolic sink for lipids, glucose, and branched-chain amino acids. Enhanced BAT thermogenesis is thought to tightly couple with beneficial energy metabolism. However, in this issue of the *JCI*, Huang et al. report a mouse model in which BAT thermogenesis was impaired, yet systemic glucose and lipid homeostasis were improved, on a high-fat diet compared with what occurred in control mice. The authors showed that BAT-specific deletion of mitochondrial thioredoxin-2 (TRX2) impaired adaptive thermogenesis through elevated mitochondrial reactive oxygen species (ROS) and cytosolic efflux of mitochondrial DNA. On the other hand, TRX2 loss enhanced lipid uptake in the BAT and protected mice from obesity, hypertriglyceridemia, and insulin resistance. This study provides a unique model in which BAT does not require thermogenesis per se to function as a lipid sink that leads to metabolic benefits in vivo.

## Linking BAT thermogenesis and systemic energy homeostasis

Brown and beige adipocytes harbor multilocular lipid droplets, possess a high number of mitochondria, and dissipate energy in the form of heat (1). Along with the ability to burn calories, brown adipose tissue (BAT) functions as a substantial metabolic sink for lipids, glucose, and branched-chain amino acids (2–4). Following cold exposure and subsequent activation of the β-3 adrenergic receptor (β3-AR), lipolysis in white adipose tissue (WAT), including the liberation of triglycerides (TG) to glycerol and free fatty acids (FFAs), triggers the activation of BAT thermogenesis (5).

Accordingly, the long-standing consensus in the field has been that enhanced BAT thermogenesis is closely linked to improved glucose tolerance, insulin sensitivity, and lipid profiles. In support of this notion, genetic ablation of brown and beige fat, for example, by BAT-specific overexpression of the diphtheria toxin or deletion of the central transcription factor, such as euchromatic histone-lysine *N*-methyltransferase 1 (EHMT1), causes obesity, insulin resistance, glucose intolerance, and dyslipidemia (6, 7).

## UCP1 thermogenesis and fuel uptake in BAT

Uncoupling protein 1 (UCP1) is a proton carrier that is selectively expressed in brown adipocytes and localizes to the mitochondrial inner membrane. UCP1 is required for BAT thermogenesis, as determined by genetic deletion of UCP1, which disrupts mitochondrial proton uncoupling in brown adipocytes and causes severe thermogenic defects following cold exposure in mice (8). However, UCP1-KO mice are not obese unless kept under a thermoneutral condition, and the metabolic phenotype does not recapitulate that found in brown and beige fat-ablation mice (6, 7, 9).

Notably, UCP1-mediated thermogenic activity does not reflect the fuel uptake of brown and beige fat. For instance, genetic deletion of UCP1 did not affect glucose uptake rates in brown and beige fat following activation of the β3-AR pathway (10–12). Chronic treatment with a β3-AR agonist (CL316,243) was able to stimulate glucose uptake in the interscapular BAT depots and improve systemic glucose tolerance in UCP1-KO mice on a high-fat diet (11). Also, fatty acid uptake into brown and beige fat was further enhanced in UCP1-KO mice compared with wild-type mice in response to β3-AR agonist treatment (13). Importantly, enhanced brown and beige fat biogenesis leads to improved insulin sensitivity, glucose tolerance, and lipid homeostasis, even in the absence of UCP1 (12). These studies demonstrate that UCP1 is required for brown fat thermogenesis, but dispensable for fuel-uptake capacity in brown and beige fat. This conclusion is somewhat surprising, but suggests a possibility that BAT thermogenesis does not necessarily couple with metabolic improvement at the organismal level.

## Mitochondrial inflammation and the role of TRX2

Mitochondria play a key role in oxidative stress and immune response through the regulation of mitochondrial reactive oxygen species (mtROS), mitochondrial DNA (mtDNA), and antioxidant systems (14). Whereas mtROS serve as a key signaling entity in the mitochondria, uncontrolled mtROS induce oxidative stress and mtDNA release to the cytosol. Excess mtROS production and cytosolic mtDNA are known to activate the NLRP3 inflammasome via cyclic GMP/AMP synthase/stimulator of IFN genes (cGAS/STING), leading to elevated production of proinflammatory cytokines and inflammation (14, 15).

As a defense mechanism to protect cells against mitochondrial oxidative stress, mtROS is rapidly neutralized by the mitochondrial thioredoxin system that is composed of thioredoxin-2 (TRX2), TRX2 reductase (TRXR2), and peroxiredoxin 3 (PRX3). Of these, TRX2 is an essential mitochondrial antioxidant that scavenges ROS via mitochondrial peroxidase PRX3 (16). It has been demonstrated that overexpression of TRX2 suppresses inflammatory pathways and apoptosis by reducing mtROS production (17). Conversely, TRX2 loss leads to the accumulation of intracellular ROS and apoptotic cell death (18).

## Uncoupling of thermogenic capacity and metabolic benefit

**Figure 1:**
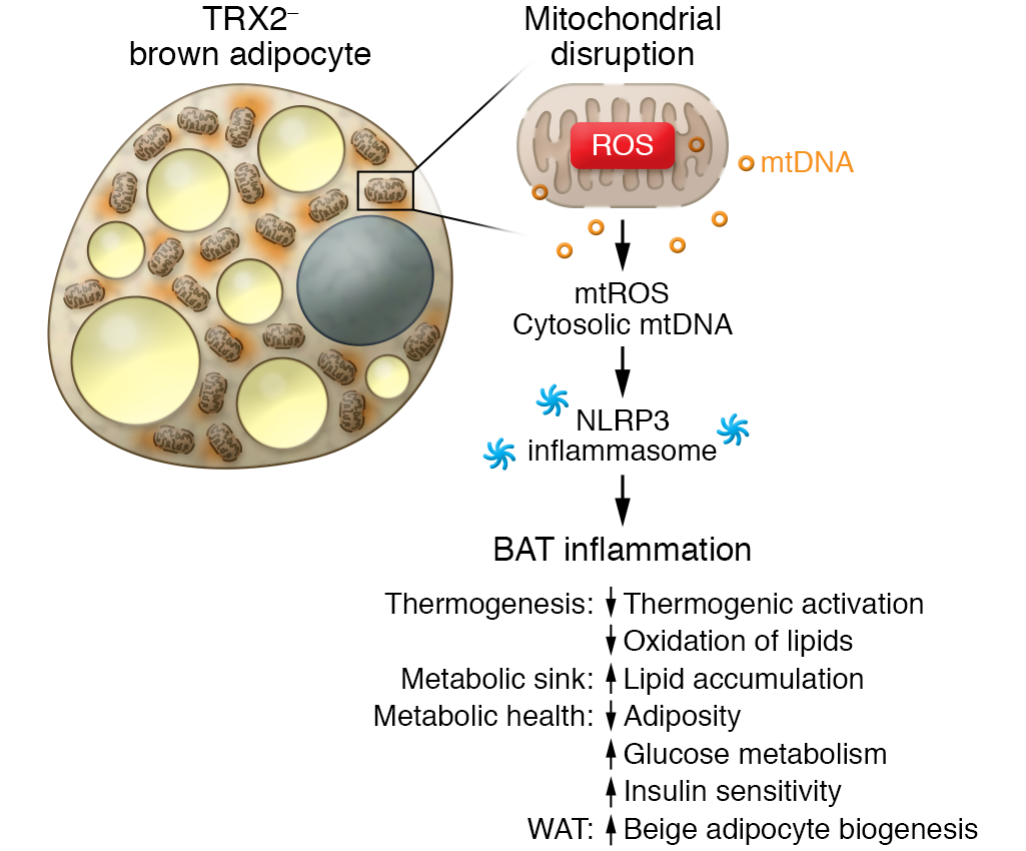
BAT improves systemic metabolism by enhancing lipid clearance from the circulation, regardless of impaired thermogenic activity. Brown adipocytes harbor multilocular lipid droplets, possess a high number of mitochondria, and dissipate energy in the form of heat. Huang et al. (19) showed that BAT-specific TRX2 deficiency caused BAT inflammation through activation of the NLRP3 inflammasome pathway. A mitochondrial defect enhanced lipid uptake into brown adipocytes, leading to improved systemic glucose and lipid homeostasis even though BAT thermogenesis was impaired.

In this issue of the *JCI*, Huang et al. asked how mitochondrial inflammation in BAT influenced systemic energy balance and glucose homeostasis in mice. To this end, the authors generated a mouse model in which TRX2 was deleted in a BAT-specific manner by using *UCP1-Cre*, herein called *Trx2^BATKO^* mice (19). The authors found that BAT-specific TRX2 deletion increased mtROS production and cytosolic mtDNA, leading to disrupted mitochondrial integrity. These molecular changes in the BAT activated mitochondrial immune responses, including the cGAS/STING and NLRP3 inflammasome pathways. Furthermore, adaptive thermogenesis and fatty acid oxidation in the BAT were substantially impaired in *Trx2^BATKO^* mice. However, the authors found that lipid uptake into BAT was enhanced in *Trx2^BATKO^* mice relative to control mice. The enhanced lipid uptake into BAT was accompanied by reduced adiposity and plasma TG levels as well as improved glucose and insulin sensitivity on a high-fat diet (Figure 1).

To probe the underlying mechanism, Huang et al. next asked whether enhanced inflammasome activation in the BAT of *Trx2^BATKO^* mice was responsible for the metabolic phenotypes. To address this question, the authors treated *Trx2^BATKO^* mice with MCC950, a pharmacological inhibitor of the NLRP3 inflammasome. MCC950 treatment effectively ameliorated BAT thermogenic function and reversed metabolic benefits, resulting in increased body weight and impaired glucose homeostasis. These data provide compelling evidence that mitochondrial inflammation in BAT profoundly influences thermogenesis and whole-body energy homeostasis (19).

## Future perspectives

How does a defect in BAT thermogenesis lead to metabolic benefits? One intriguing observation in the study by Huang et al. was enhanced beige fat biogenesis in the inguinal WAT of *Trx2^BATKO^* mice (19). This result aligns with a previous study showing that impaired brown adipocyte development in the interscapular BAT induces compensatory browning of WAT, specifically, beige fat biogenesis in mice (20). Huang and colleagues demonstrated that *Trx2^BATKO^* mice showed enhanced lipolysis, fatty acid oxidation, and glucose utilization in the inguinal WAT of *Trx2^BATKO^* mice following β3-AR agonist CL316,243 treatment. Conversely, inhibition of NLRP3 inflammasome by MCC950 treatment reversed the biogenesis of beige fat in *Trx2^BATKO^* mice, showing a tight association between enhanced beige fat biogenesis and improved glucose homeostasis (19).

Enhanced beige fat biogenesis in the inguinal WAT was insufficient to rescue the hypothermic phenotype of *Trx2^BATKO^* mice (19). This result makes sense because the contribution of beige fat to whole-body heat production appears marginal compared with that of BAT and skeletal muscle (12). However, emerging evidence shows that beige fat biogenesis potently represses adipose tissue inflammation and fibrosis in a UCP1-independent manner (21, 22). Importantly, repression of adipose tissue fibrosis and inflammation is associated with improved insulin sensitivity (21, 22). It would be intriguing to determine the degree to which compensatory activation of beige fat biogenesis contributes to metabolic improvement in *Trx2^BATKO^* mice.

A technical hurdle to exploring this question is the lack of genetic tools for targeting a selective adipose tissue depot in vivo. Adiponectin-*Cre* has been widely used to delete a gene of interest in all differentiated adipocytes, including brown, beige, and white adipocytes. In contrast, *UCP1-Cre* targets UCP1-expressing cells, primarily in brown adipocytes and to a much lesser extent in beige adipocytes. Of note, a recent paper reported that *UCP1-Cre* also targeted the hypothalamus, thymus, adrenal glands, and kidney, raising a possibility that *UCP1-Cre* might alter neuronal activity if a gene of interest was expressed in these *UCP1-Cre^+^* nerves (23). Thus, developing tools that enable us to manipulate specific adipose cells and/or depots would open up opportunities to dissect the functional relationship between thermogenesis and metabolic benefit that is associated with enhanced brown and beige fat biogenesis.

## Version 1 05/02/2022

Electronic publication

## References

[1] Sidossis L, Kajimura S (2015). Brown and beige fat in humans: thermogenic adipocytes that control energy and glucose homeostasis. J Clin Invest.

[2] Chondronikola M (2016). Brown adipose tissue activation is linked to distinct systemic effects on lipid metabolism in humans. Cell Met.

[3] Bartelt A (2011). Brown adipose tissue activity controls triglyceride clearance. Nat Med.

[4] Yoneshiro T (2019). BCAA catabolism in brown fat controls energy homeostasis through SLC25A44. Nature.

[5] Shin H (2017). Lipolysis in brown adipocytes is not essential for cold-induced thermogenesis in mice. Cell Metab.

[6] Lowell BB (1993). Development of obesity in transgenic mice after genetic ablation of brown adipose tissue. Nature.

[7] Ohno H (2013). EHMT1 controls brown adipose cell fate and thermogenesis through the PRDM16 complex. Nature.

[8] Enerbäck S (1997). Mice lacking mitochondrial uncoupling protein are cold-sensitive but not obese. Nature.

[9] Feldmann HM (2009). UCP1 ablation induces obesity and abolishes diet-induced thermogenesis in mice exempt from thermal stress by living at thermoneutrality. Cell Metab.

[10] Hankir MK (2017). Dissociation between brown adipose tissue ^18^F-FDG uptake and thermogenesis in uncoupling protein 1-deficient Mice. J Nucl Med.

[11] Olsen JM (2017). β3-Adrenergically induced glucose uptake in brown adipose tissue is independent of UCP1 presence or activity: Mediation through the mTOR pathway. Mol Metab.

[12] Ikeda K (2017). UCP1-independent signaling involving SERCA2b-mediated calcium cycling regulates beige fat thermogenesis and systemic glucose homeostasis. Nat Med.

[13] Fischer AW (2020). Brown adipose tissue lipoprotein and glucose disposal is not determined by thermogenesis in uncoupling protein 1-deficient mice. J Lipid Res.

[14] Angajala A (2018). Diverse roles of mitochondria in immune responses: novel insights into immuno-metabolism. Front Immunol.

[15] Ishikawa H, Barber GN (2008). STING is an endoplasmic reticulum adaptor that facilitates innate immune signalling. Nature.

[16] Chae HZ (1999). Characterization of three isoforms of mammalian peroxiredoxin that reduce peroxides in the presence of thioredoxin. Diabetes Res Clin Pract.

[17] Hansen JM (2006). Mitochondrial thioredoxin-2 has a key role in determining tumor necrosis factor-α–induced reactive oxygen species generation, NF-κB activation, and apoptosis. Toxicol Sci.

[18] Tanaka T (2002). Thioredoxin-2 (TRX-2) is an essential gene regulating mitochondria-dependent apoptosis. EMBO J.

[19] Huang Y (2022). Brown adipose TRX2 deficiency activates mtDNA-NLRP3 to impair thermogenesis and protect against diet-induced insulin resistance. J Clin Invest.

[20] Schulz TJ (2013). Brown-fat paucity due to impaired BMP signalling induces compensatory browning of white fat. Nature.

[21] Hasegawa Y (2018). Repression of adipose tissue fibrosis through a PRDM16-GTF2IRD1 complex improves systemic glucose homeostasis. Cell Metab.

[22] Sun K (2014). Endotrophin triggers adipose tissue fibrosis and metabolic dysfunction. Nat Commun.

[23] Claflin KE (2022). Conditional gene targeting using UCP1-Cre mice directly targets the central nervous system beyond thermogenic adipose tissues. Mol Metab.

